# Supplementary material for: Ecological Pest Control in Alpine Ecosystems: Monitoring Asteraceae Phytophages and Developing Integrated Management Protocols in the Three River Source Region
Source: Insects. 2025 Aug 19;16(8):861. doi: 10.3390/insects16080861 (PMC12386970; doi:10.3390/insects16080861)
Supplement: Supplementary file 1 [file insects-16-00861-s001.zip › Additional Figure information’s.pdf]

Additional Figure information's

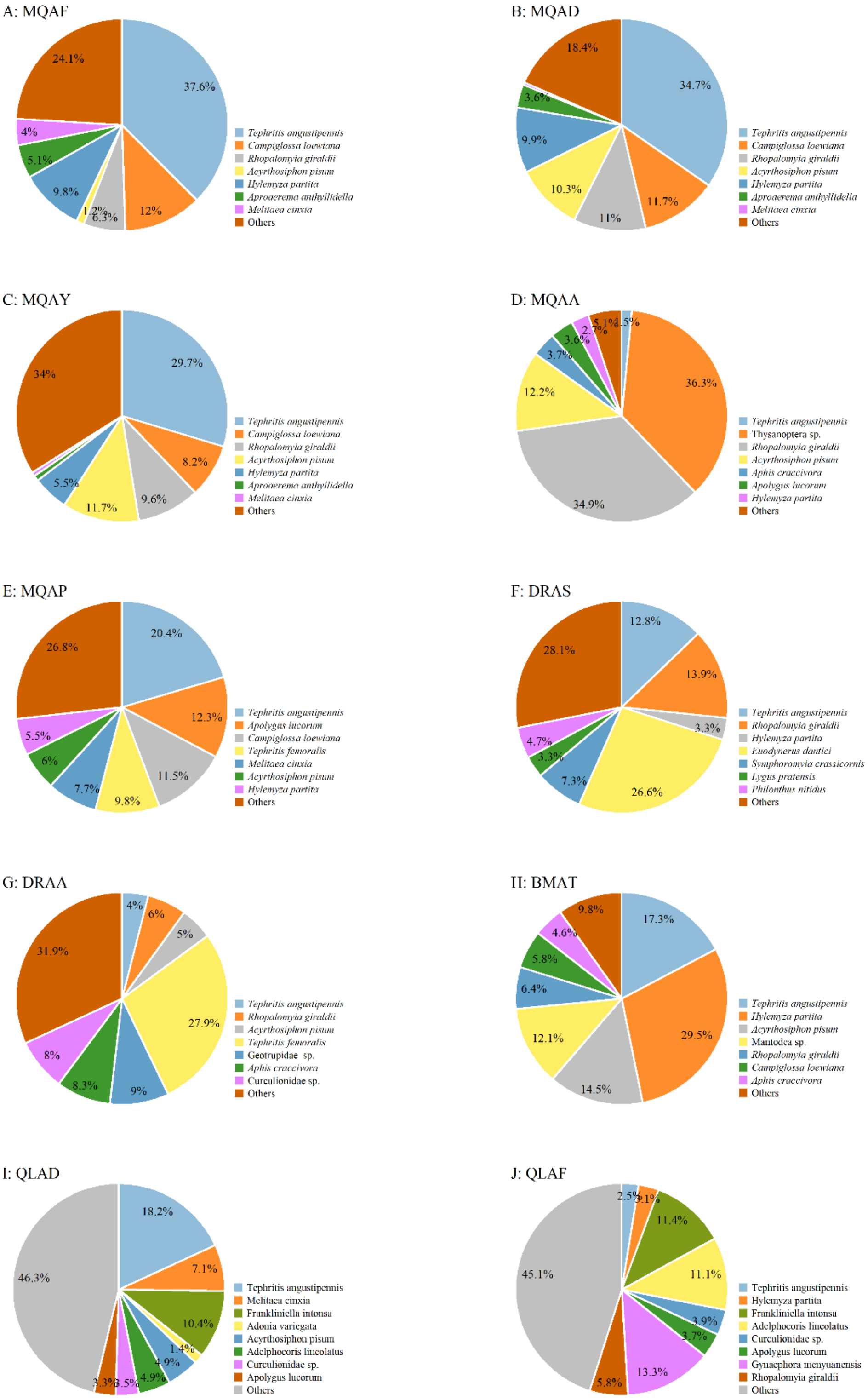

Figure S1 Percentage of dominant species in different sampling sites.

Noye: A-J represent the proportion of dominant pest species on MQAF, MQAD, MQAY, MQAA, MQAP, DRAS, DRAA, BMAT, QLAD and QLAF, respectively.

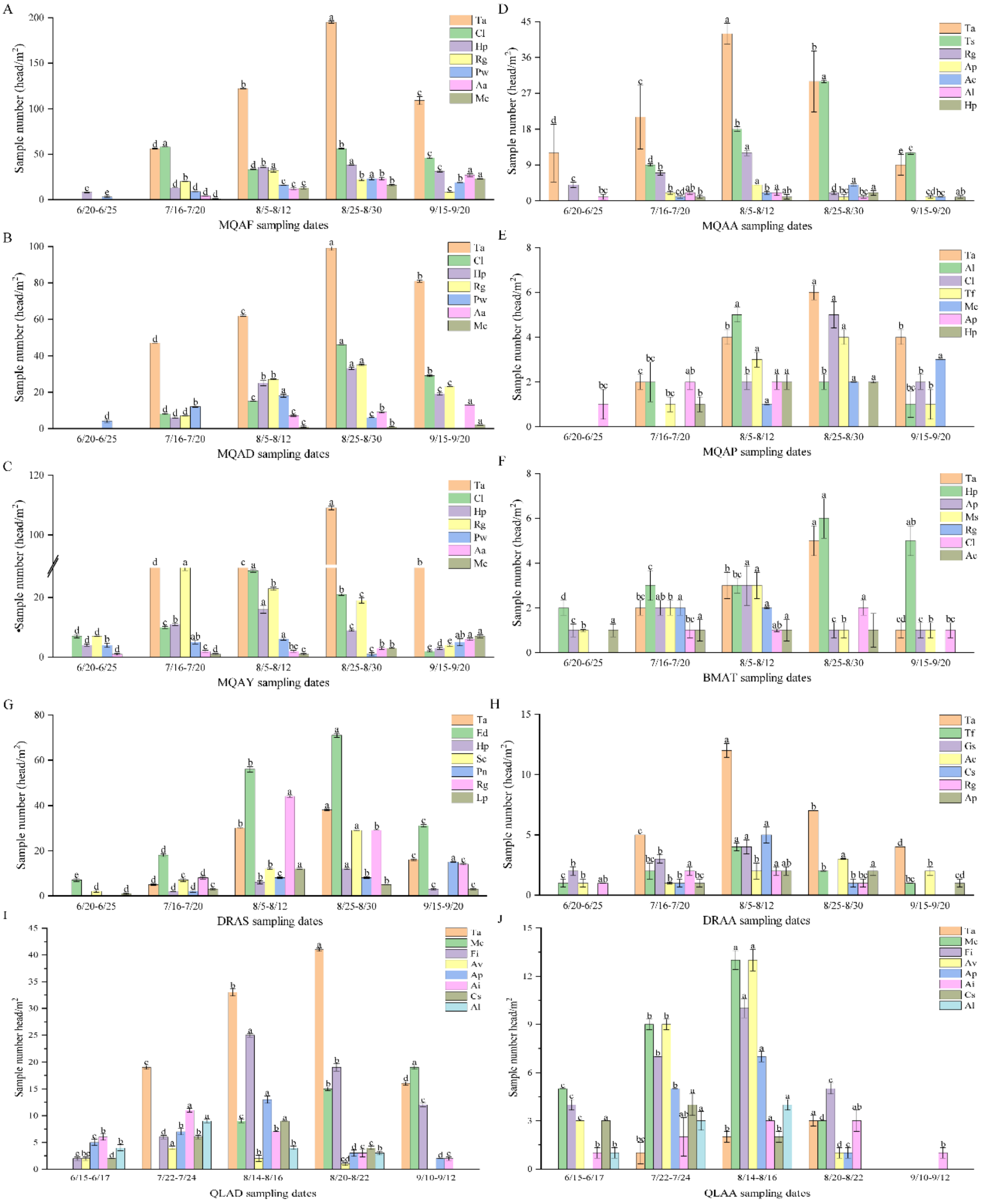

Figure S2 Temporal dynamics of insect dominant species in all sampling sites.

Note: Lowercase letters next to the numbers indicate the variability of population comparisons of the same pest at different times; identical letters indicate no significant difference ($P > 0.05$), while different letters denote a statistically significant difference ($P < 0.05$). The species abbreviations in the figure represent the following: *Tephritis angustipennis* (Ta), *Melitaea cinxia* (Mc), *Frankliniella intonsa* (Fi), *Adelphocoris lineolatus* (Ai), Curculionidae sp. (Cs), *Adonia variegata* (Av), *Apolygus lucorum* (Al), *Acyrthosiphon pisum* (Ap), *Gynaephora menyuanensis* (Gm), *Rhopalomyia giraldii*

(Rg), *Hylemyza partita* (Hp), *Campiglossa loewiana* (Cl), *Parasitoid wasp* (Pw), *Aproaerema anthyllidella* (Aa), Thysanoptera sp. (Ts), *Aphis craccivora* (Ac), *Tephritis femoralis* (Tf), *Euodynerus dantici* (Ed), *Symphoromyia crassicornis* (Sc), *Philonthus nitidus* (Pn), *Lygus pratensis* (Lp), and Geotrupidae sp. (Gs). The same as below.

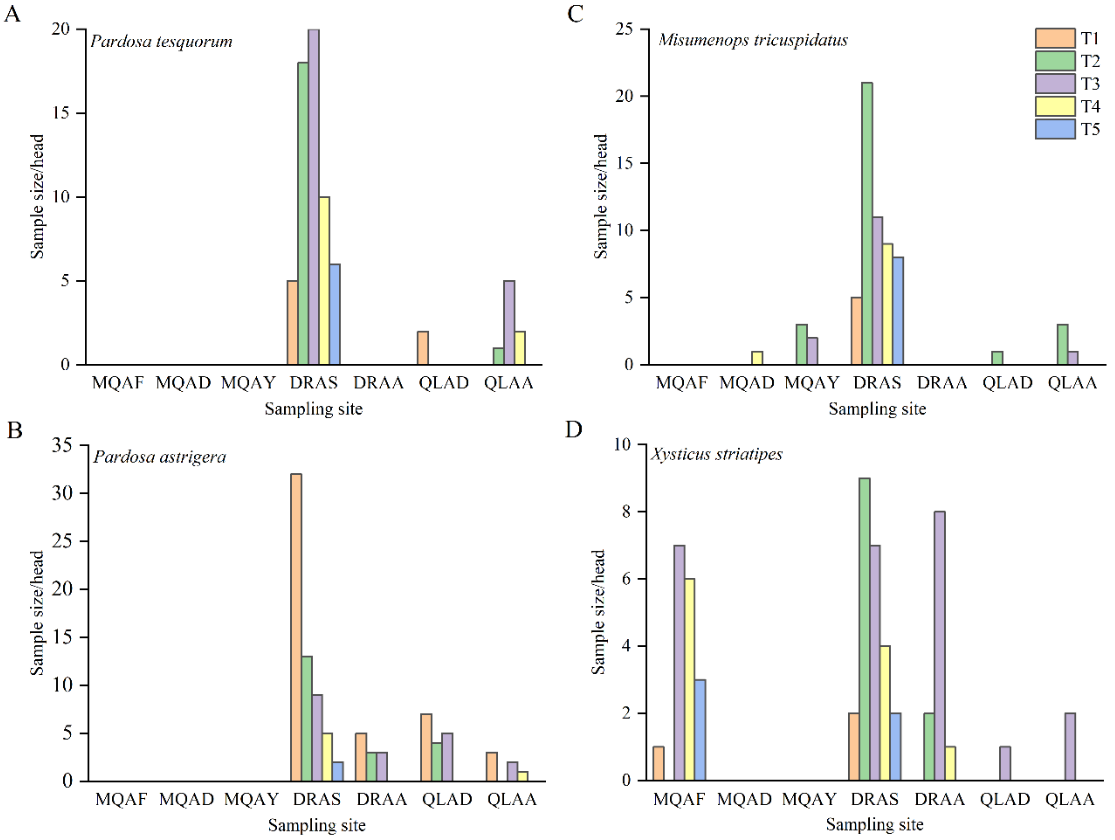

Figure S3 Occurrence dynamics of dominant spiders at each sampling site.

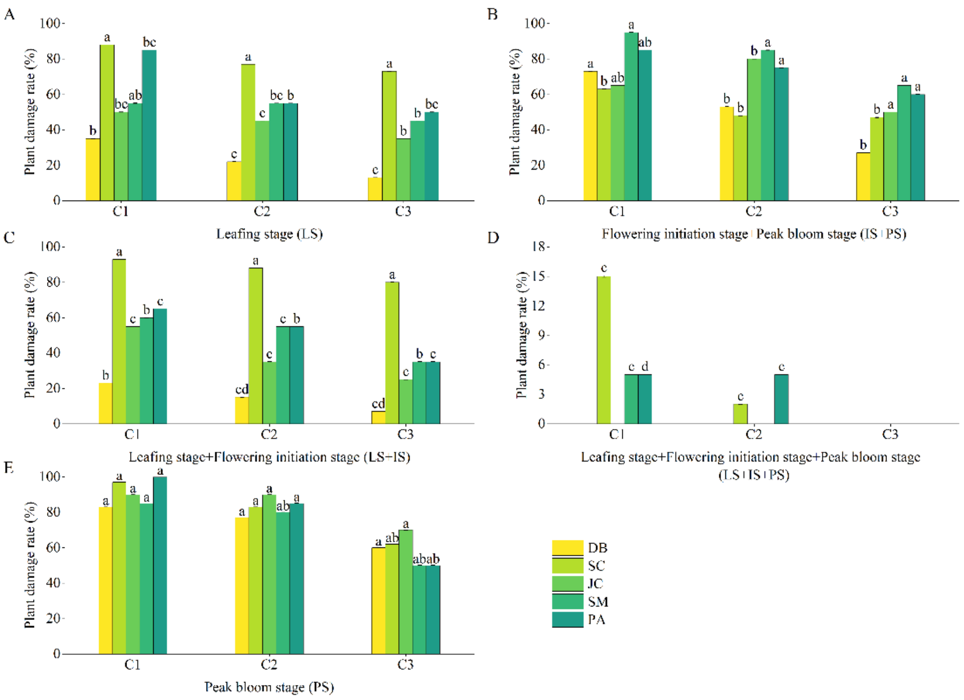

Figure S4 Comparison of the plant damage rate (PDR) of different pesticide concentrations applied at various times.

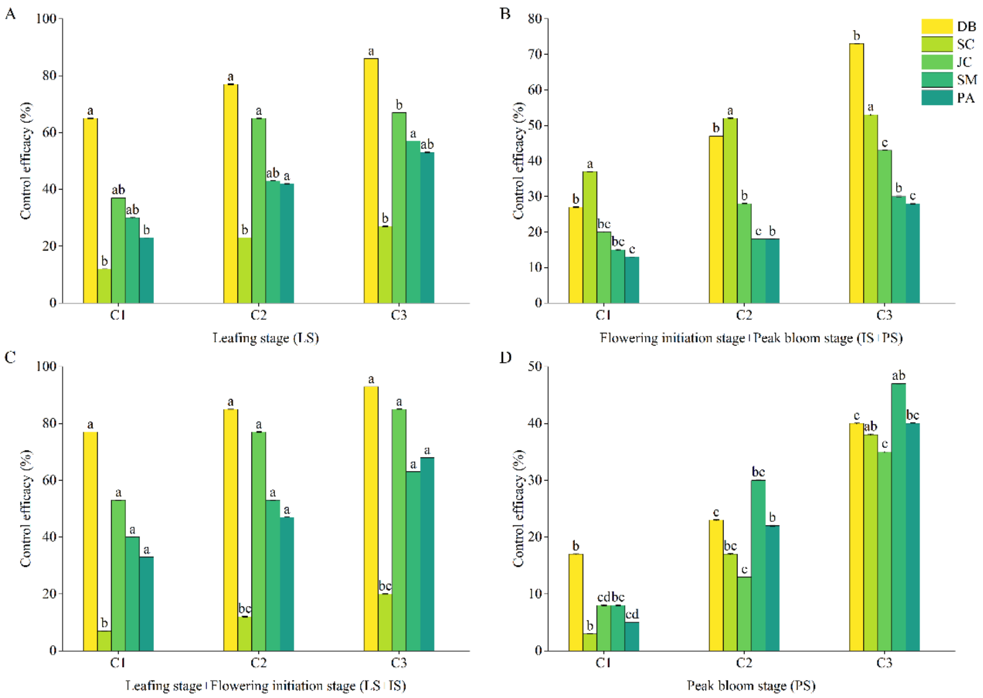

Figure S5 Comparison of the control efficacy (CE) of different pesticide concentrations applied at various times.

Note: There were almost no pests after three rounds of spraying, so no data analysis chart for this treatment is shown. The same as below.

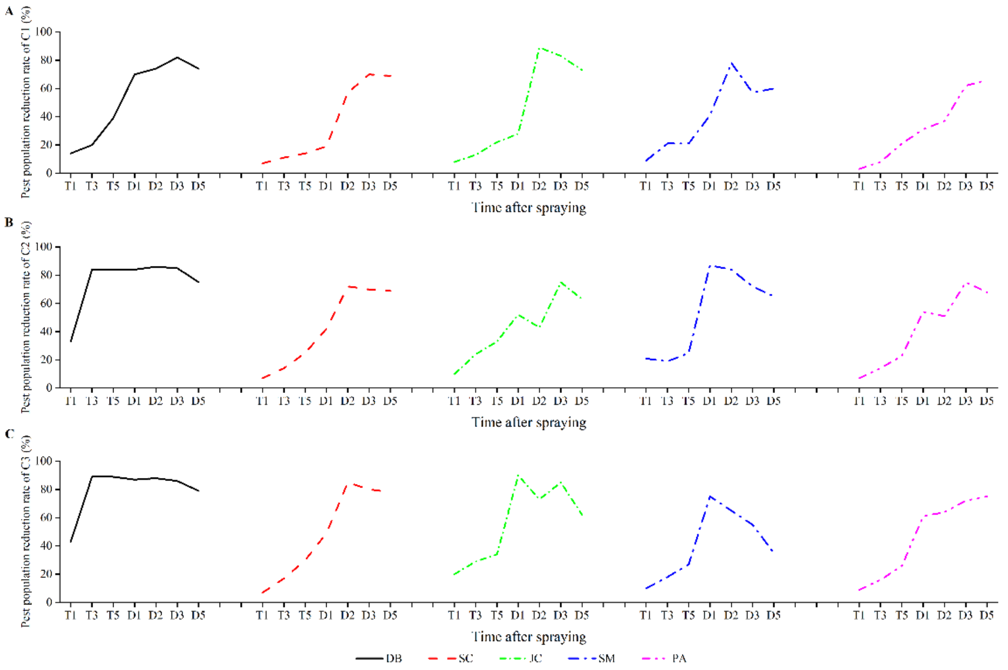

Figure S6 Comparison of pest population reduction rate (PRR) at different time periods after applying pesticides at various concentrations.

Note: A-C represent the rate of decline in the pest population density at concentrations C1, C2, and C3, respectively.

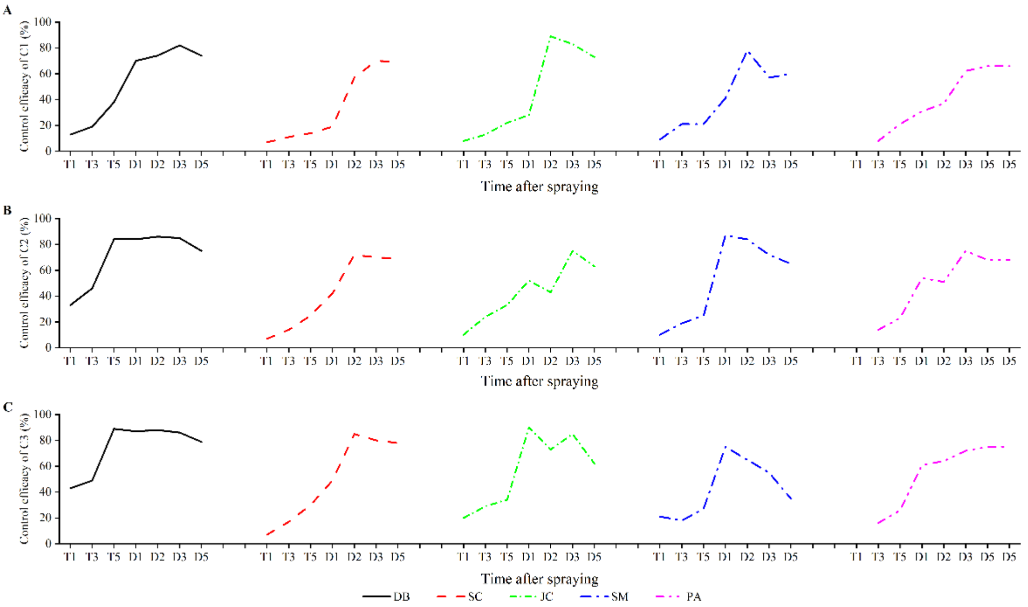

Figure S7 Comparison of control efficacy (CE) at different time periods after applying pesticides at various concentrations.

Note: A-C represent the control efficacy at concentrations C1, C2, and C3, respectively.
