# Supplementary figures and images for: Ecological Pest Control in Alpine Ecosystems: Monitoring Asteraceae Phytophages and Developing Integrated Management Protocols in the Three River Source Region
Source: Insects. 2025 Aug 19;16(8):861. doi: 10.3390/insects16080861 (PMC12386970; doi:10.3390/insects16080861)

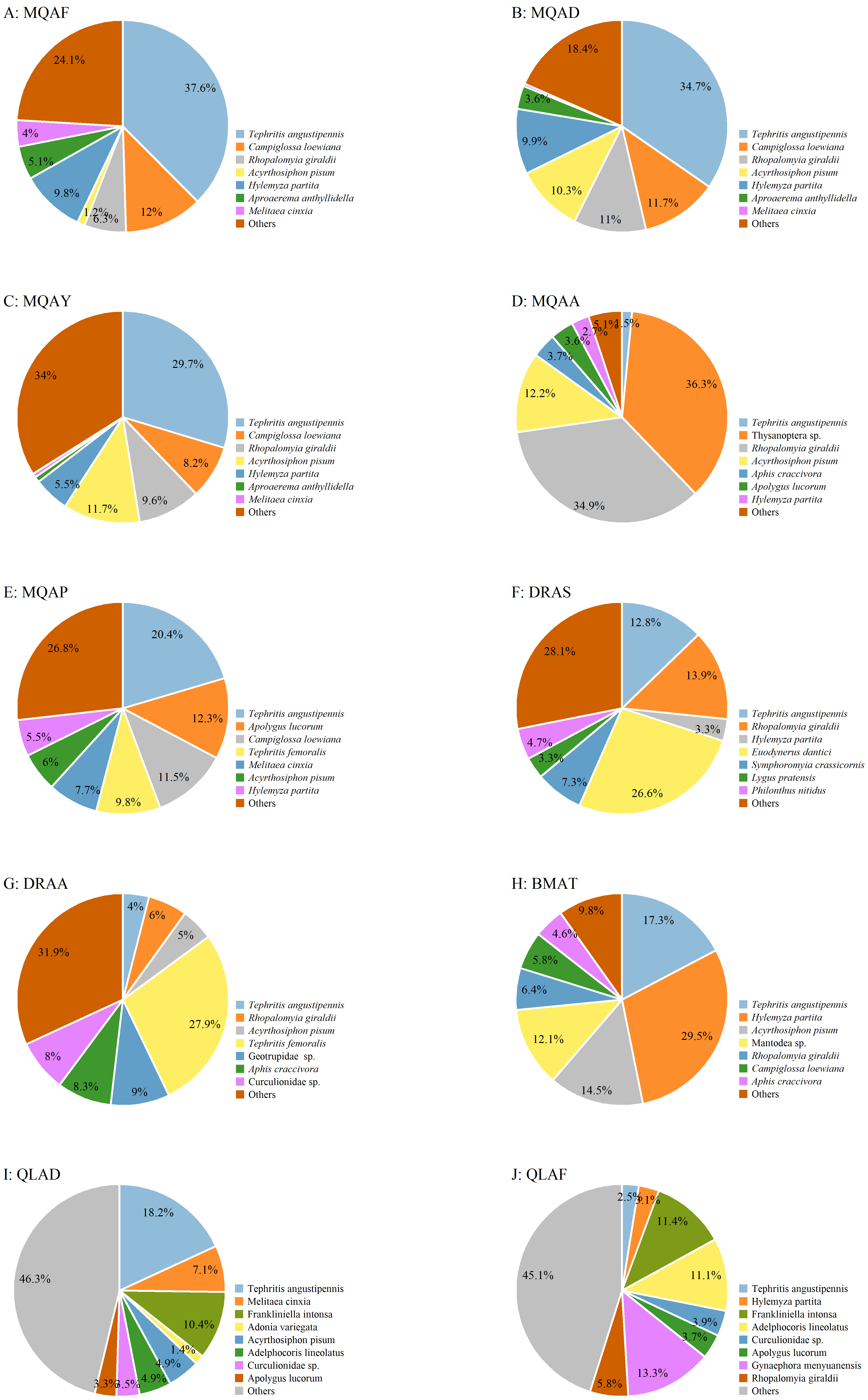

Supplement: Supplementary file 1 [file insects-16-00861-s001.zip › Figure S1.tif]

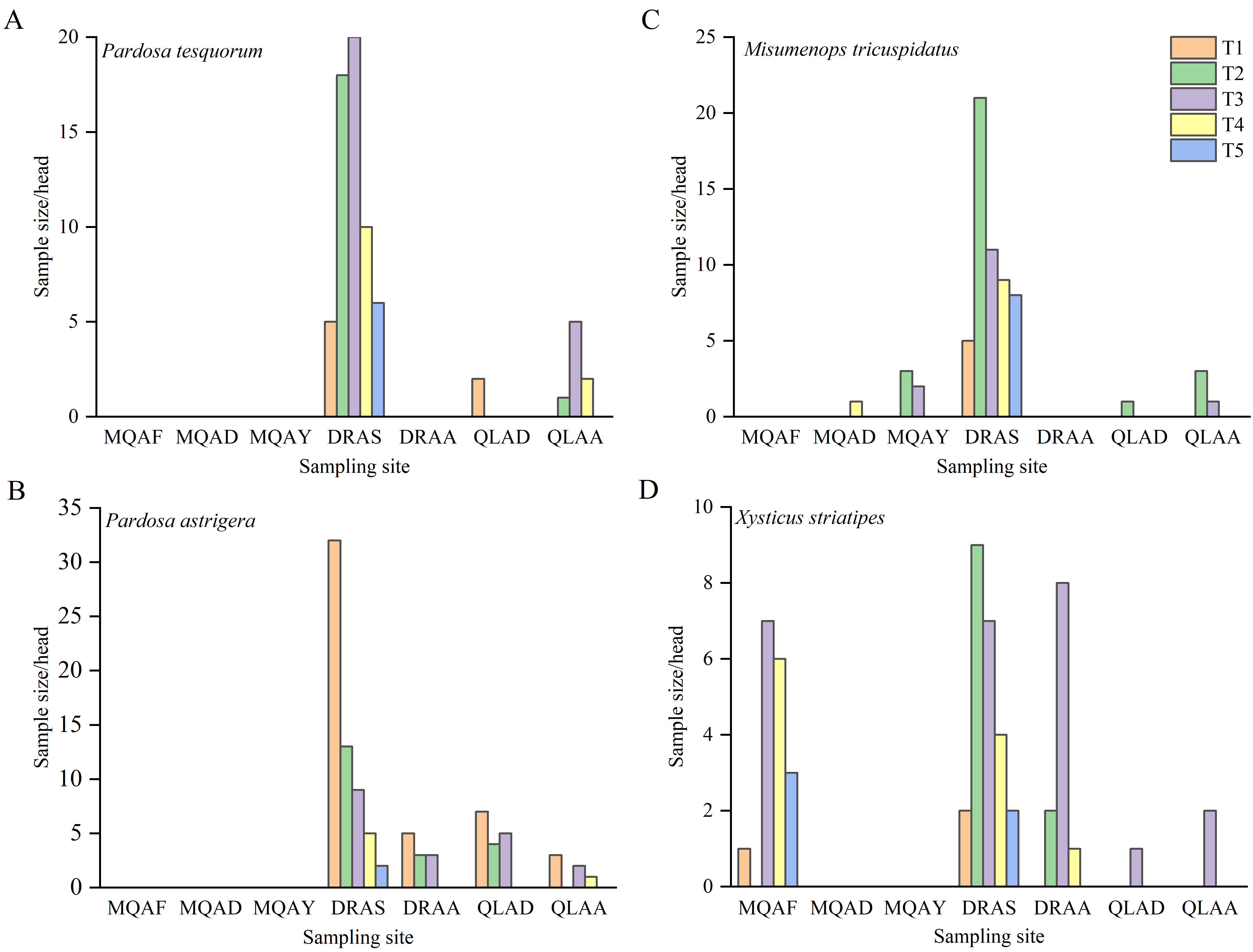

Supplement: Supplementary file 1 [file insects-16-00861-s001.zip › Figure S3.tif]

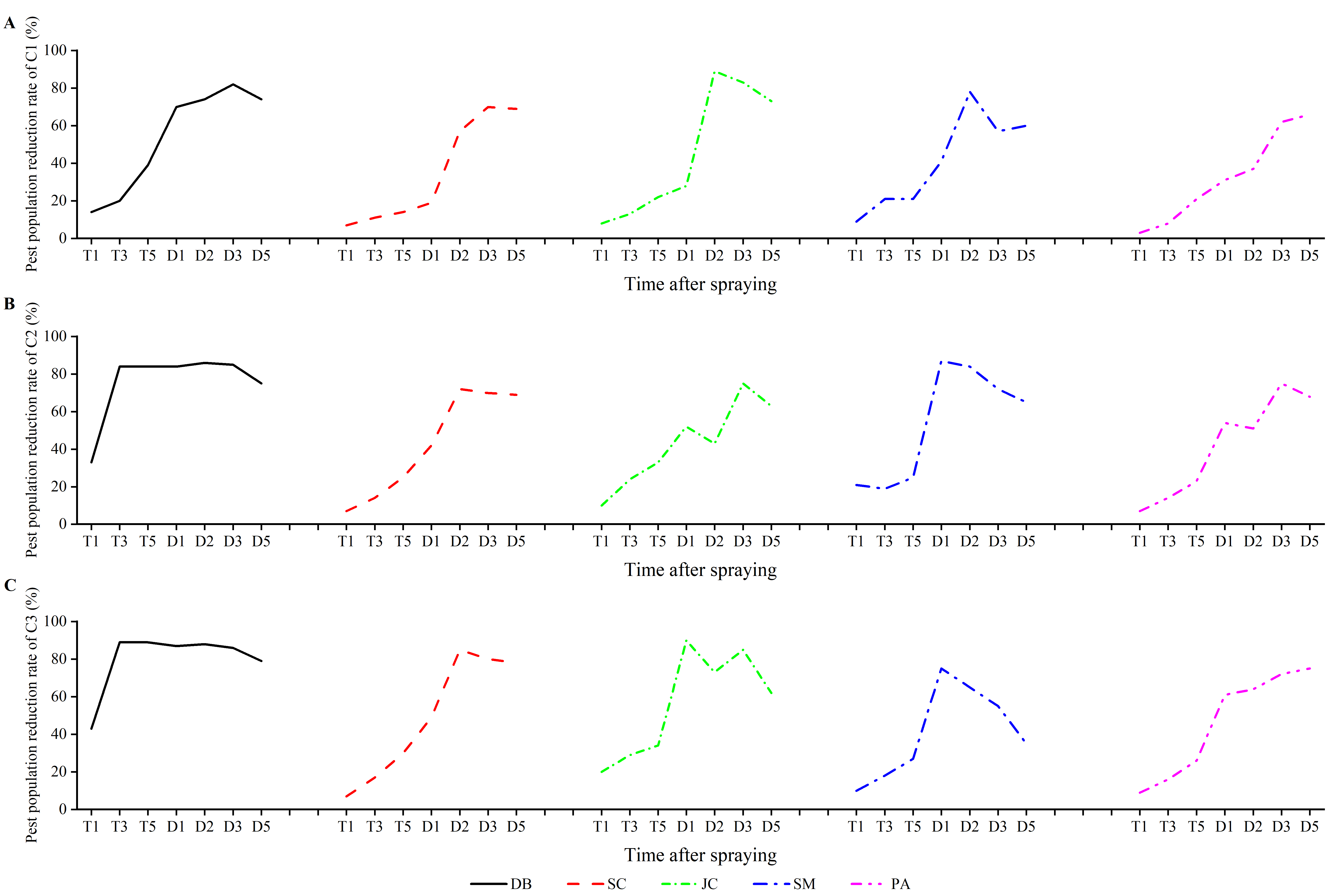

Supplement: Supplementary file 1 [file insects-16-00861-s001.zip › Figure S6.tif]

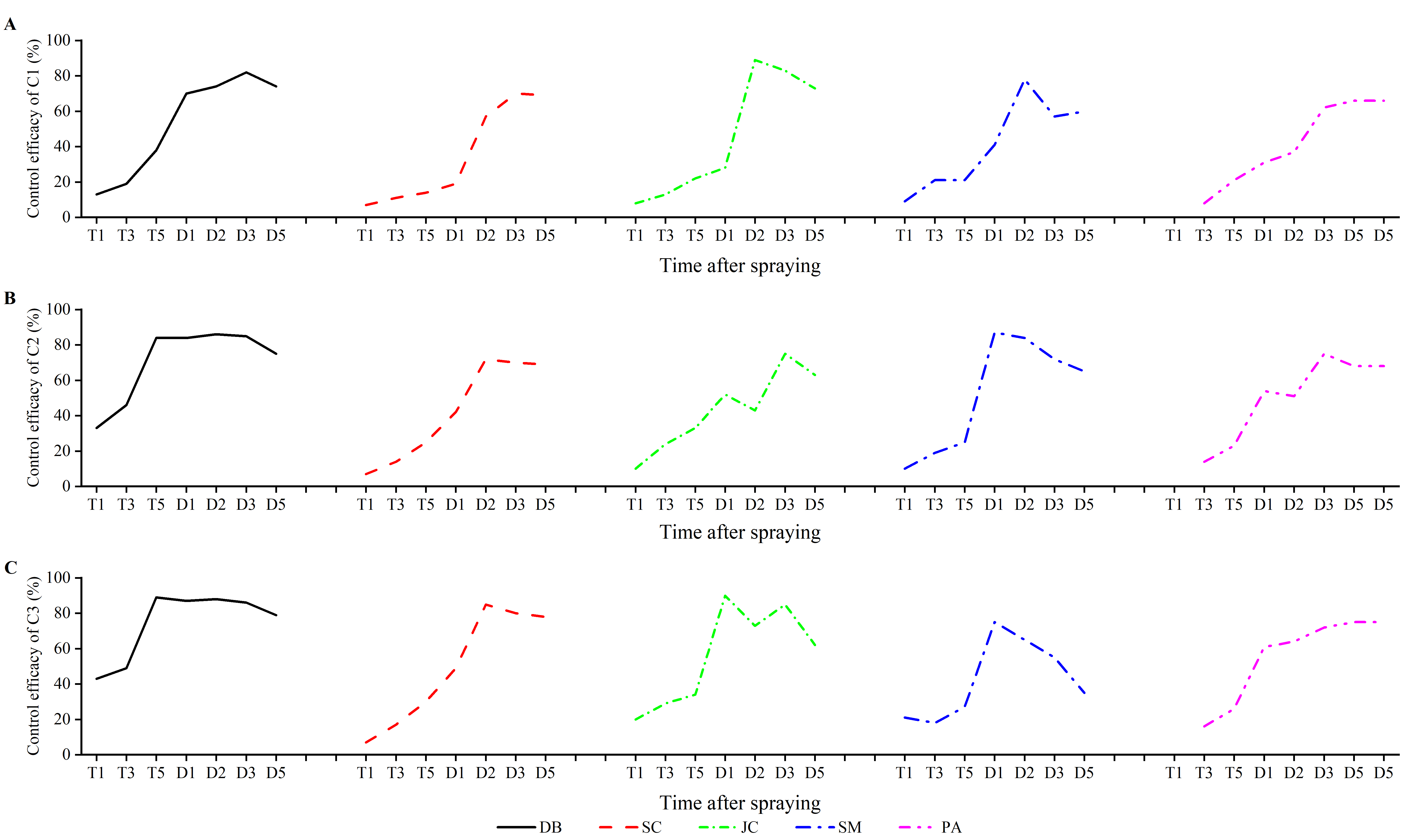

Supplement: Supplementary file 1 [file insects-16-00861-s001.zip › Figure S7.tif]
